# The Mansion House Dinner

**Published:** 1898-05-14

**Authors:** 


The Hospital

A Journal of

The Medical Sciences and Hospital Administration.

Vol. XXIV.—No. 607.] Edited by Sir Henry Burdett, K.C.B., and Solomon C. Smith, M.D., M.R.C.P. [May 14, 1898.

### The Mansion House Dinner.

The banquet given last week by the Lord Mayor to the medical profession was not only noteworthy as the first instance of such an entertainment, but also as having afforded an opportunity to the Secretary of State for War to make announcements with regard to the future position of the officers of the Army Medical Department which were exceedingly gratifying to his hearers. The invitations, which were to meet the Presidents of the Royal Colleges of Physicians and Surgeons, were issued at a time when the presidency of the General Medical Council was vacant; and it is probable that Sir William Turner had accepted, as a distinguished surgeon and teacher, before his election to fill the vacancy. But for this, the opportunity would no doubt have been taken to settle the question of precedence between the President of the Council and the President of the College of Physicians; a question on which the late Sir Andrew Clark felt very strongly, and which has not yet received an authoritative decision from the officials at the College of Arms. It is thought by some that the greater antiquity of the College of Physicians should give the first place to its President, and by others that this place is due to the President of the Council, as the holder of an office created by Act of Parliament, and as the head of a body which, in certain questions, is appointed to exercise control over the Colleges. It is quite conceivable that, in the absence of any official declaration upon the point, circumstances might arise in which each President would feel it almost his duty to maintain what might prove to be a rightful privilege of his office. If, as we hope may be the case, the example of hospitality just set is followed in future years, it would be very desirable for one or other of the corporations concerned to take steps for the purpose of determining the question.

We do not know how far the fact that Lord Lansdowne had so pleasant a message to deliver may have been the explanation of his presence at the banquet, but it is at least certain that no more favourable occasion could have been found for the announcement which he made, and which was rendered doubly acceptable to his audience by the frank and ungrudging recognition of the merits as well as of the policy of the case. We publish a report of his speech in another column, and so need not here refer to it in detail. It is only too notorious, to all interested in the question, that the Army Medical Department, for some years past, has been in a state of very imperfect efficiency, weak in point of numbers, and unable to attract the better class of the students who were annually leaving the schools of medicine. The old regimental system, which, in time of peace, was the best for everybody concerned, broke down utterly in time of war, when it is of the first consequence that surgeons should be independent of, or at least freely detachable from, their regiments, and should be available for duty wherever they may be most required. The fulfilment of this condition implies another; because it imposes upon the chief medical officer of such a corps as is now necessary the duty of so organising and marshalling his forces as to have them ready where they are wanted. The forces in question comprise not only surgeons, but a large body of drilled soldiers, serving in various capacities as aids to the sick and wounded, as bearers, dispensers, drivers, hospital orderlies, and the like—men who, in the field, would recognise only military authority, and would yield prompt and unquestioning obedience only to military rank. When a regimental surgeon went into action with his regiment every man in it knew him, and was ready to carry out his instructions; but, when an officer of the Medical Department goes into action with men who have never seen him before, he must, if he is to command them, be clothed with the authority which nothing but recognised military rank will give. This elementary truth has at last been recognised by the War Office, and has been recognised with a fulness which leaves nothing to be desired. The officers of the Medical Department are to be to all intents and purposes military officers, and not civilians wearing military uniform; and, until they reach the rank of general, the prefix surgeon will fall away from their titles. The next war will show whether its retention in the case of surgeons-general will be attended by any practical inconvenience; and, if so, there can be no doubt that it will be speedily abandoned. With this one exception, the profession has now been promised everything that its members can reasonably ask, and there can be no doubt that the social grievances which have been greatly felt in the service will disappear in the wake of the official ones. It would be a fitting crown to Lord Lansdowne's good work if he could arrange for a return to something like the regimental system in time of peace, say by appointing an officer to a particular regiment for a term of years, unless the exigencies of the service imperatively demanded his removal. If this were done it would at once restore to the surgeon his "home" in the regiment, would bring back the old *camaraderie* between the combatant and the medical officers, and, what is of very great imtance to the welfare of the service, would restore not only the position of the doctor, but also the position of the doctors' wife.

